# Interstitial Lung Disease in Werner Syndrome: A Case Report of a 55-Year-Old Male Patient

**DOI:** 10.1155/2015/361694

**Published:** 2015-12-16

**Authors:** Tiphaine Goletto, Flora Crockett, Selim Aractingi, Cecile Toper, Patricia Senet, Jacques Cadranel, Jean-Marc Naccache

**Affiliations:** ^1^Service de Pneumologie, Hôpital Universitaire Tenon, 75020 Paris, France; ^2^Service de Dermatologie, Hôpital Universitaire Cochin, 75014 Paris, France; ^3^Service de Dermatologie, Hôpital Universitaire Tenon, 75020 Paris, France

## Abstract

Werner syndrome (WS) is a progeroid or premature aging syndrome characterized by early onset of age-related pathologies and cancer. The average life expectancy of affected people is 52.8 years and tends to increase. The major causes of death are malignancy and myocardial infarction. Increased telomere attrition and decay are thought to play a causative role in the clinical and pathological manifestations of the disease. Although telomere length, with or without germline mutation, is known to be associated with interstitial lung disease, the latter is not associated with WS. To the best of our knowledge, we report the first case describing a WS patient with fatal ILD. This case suggests that older patients with WS could develop ILD. Clinical outcome of WS patients may thus be improved by counselling them regarding smoking cessation or other exposure and by proposing antifibrotic therapy.

## 1. Introduction

Werner syndrome (WS) is a progeroid or premature aging syndrome characterized by early onset of age-related pathologies and cancer. Symptoms of WS manifest after 10 years, but the diagnosis is often established after 30 years. Diagnosis of WS is based on the presence of 3 among the following 4 criteria under age 35: (i) characteristic stature including short stature and light body weight, (ii) premature senescence, (iii) scleroderma-like skin changes, and (iv) endocrine-metabolic abnormalities: diabetes mellitus (DM) and hypogonadism [1]. Moreover, WS has been classified as a member of hereditary cancer-prone syndrome. The average life expectancy of affected people is 52.8 years [2]. The major causes of death are malignancy, including lung cancer, and myocardial infarction. Increased telomere attrition and decay are thought to play a causative role in the clinical and pathological manifestations of the disease. Although telomere length, with or without germline mutation in telomerase genes (TERC or TERT), is known to be associated with interstitial lung disease (ILD), the latter is not associated with WS [3]. To the best of our knowledge, this is the first case report describing a WS patient with ILD.

## 2. Patient Case

A 55-year-old patient was admitted in March 2012 for the exploration of an interstitial lung disease and a pulmonary nodule. He was of Indian origin and had lived in France since 1980. In his family history, his sister living in UK had sarcoidosis. He was a former smoker (from age 10 to 30; 40 PA) and used to work in trade clothing without any specific exposure. His medical history was noteworthy for a WS diagnosed in 2002 with all criteria cited above and mutation in WRN gene (1397 del A). His main manifestation was a major atherosclerosis with ankles cutaneous ulcerations and right calcaneus osteitis. His medication included atorvastatin, salicylic acid, and pregabalin.

**Figure 1:**
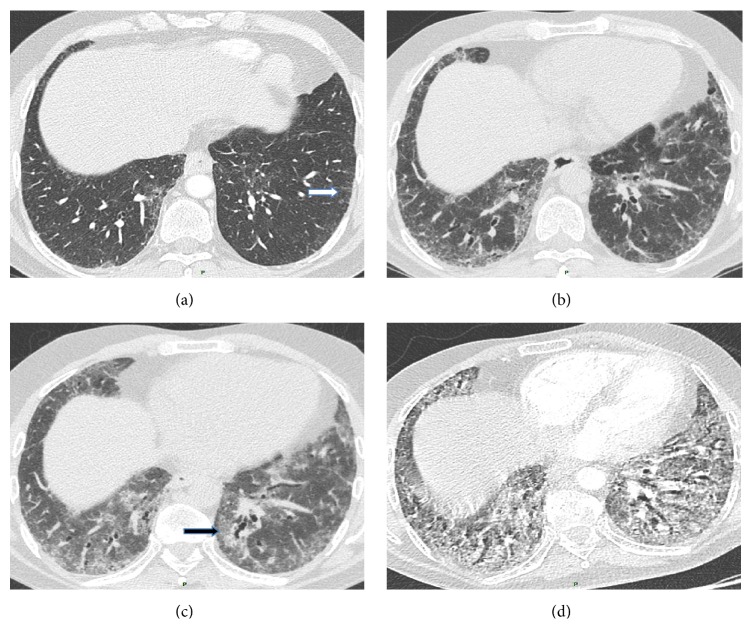
(a) HRCT in 2007 showed slight subpleural lines (arrow). (b) HRCT in March 2012 showed a predominance of subpleural reticular abnormalities with some ground glass features. (c) HRCT in July 2012 showed a worsening of reticular features with new ground glass opacities (arrow). (d) HRCT in August 2012 showed diffuse ground glass opacities with traction bronchiectasis.

He complained of dyspnea that had worsened over the last four months, classified NYHA class III at the time of evaluation. Physical examination showed bilateral basal Velcro crackles but no digital clubbing and no extrathoracic symptom suggestive of connective tissue disease. Chest CT scan revealed reticular abnormalities and traction bronchiectasis with subpleural basal predominance. There was no honeycombing. The chest CT also showed a right upper lobe nodule of 1 cm in diameter (Figure 1(b)). Discrete subpleural reticular features already existed in 2007 (Figure 1(a)). Pulmonary function test (PFT) with references values of Caucasian population showed a restrictive ventilatory defect with FVC of 1.65 L (47.4%) and DLCO of 40%. Arterial blood gas results were within normal range. Peripheral blood count and renal and liver functions were normal. Serologic testing for connective tissue disease (rheumatoid factor, anti-cyclic citrullinated peptide, anti-nuclear, anti-extractable nuclear antigen, and anti-aminoacyl tRNA synthetase antibodies) was negative. Telomerase gene mutation screening was negative. Bronchoalveolar lavage fluid analysis showed 440.000 cells/mL, 74.5% of macrophages, 12% of lymphocytes, 9% of neutrophils, and 4.5% of eosinophils. PET/CT scan did not reveal any hypermetabolism of the nodule.

Initially, a prolonged antibiotic regimen was administered as treatment for the pulmonary nodule and it was decided with regard to the ILD to stop the atorvastatin and monitor the course of the disease. The patient was considered too fragile to perform lung biopsy.

In July 2012, dyspnea worsened and CT scan revealed increased reticular opacities with appearance of patchy ground glass opacities (Figure 1(c)). The nodule was unchanged. Pulmonary function tests showed a decrease in FVC (41%) and DLCO (30%). Bronchoscopy with bronchoalveolar lavage did not suggest any infection. Echocardiography showed normal left ventricular function and no pulmonary hypertension. Taking into account the cutaneous ulcerations and the osteitis, the patient was started on a low dose of corticosteroids (20 mg/d). One month later the patient experienced severe respiratory insufficiency with rapid worsening of CT scan abnormalities including ground glass opacities associated with traction bronchiectasis (Figure 1(d)). Death occurred despite intensive treatment with bolus of corticosteroid and cyclophosphamide.

## 3. Discussion

WS is a rare autosomal recessive disorder caused by a mutation in the WRN gene located at chromosome 8p11-12 which encodes a DNA helicase. The WRN gene is the only gene known to be associated with WS and the mutation types are numerous. The protein mutated in WS, WRN, appears to play a major role in genome stability, particularly during DNA replication and telomere metabolism. WS affects between 1/150 000 people and 1/500 000 people and most cases are sporadic. 75% of the WS patients are of Japanese origin and it is more frequent in region with high consanguinity. Our patient did not report any inbreeding in his family, nor any family member diagnosed with WS. Sequencing of WRN exons revealed a homozygous mutation.

WS shares some common features with other telomere diseases, most represented by mutation of telomerase complex (TERC and TERT), like skin hyperpigmentation, premature hair greying, myeloid disorder, and risk of cancer [1, 4, 5]. Familial and sporadic ILD are known to be associated with mutation of telomerase complex but have never been described in WS [3]. In our patient, known causes of ILD as drugs, exposure, connective tissue disease, and other genetic disorders have been excluded by medical history and biological testing. CT scan features showed a “possible UIP” pattern that could correspond to IPF [6]. This raises the question of the link between IPF and WS. Several hypotheses should be therefore discussed. IPF is an ageing disease of the lung and IPF patients are in most cases over 54 years of age including patients with a mutation of the telomerase complex [7]. The reported life expectancy of 52.8 years in WS patients could not permit observing IPF [2]. Our patient was 55 years old, an uncommon longevity in WS that could have promoted the occurrence of IPF. Cigarette smoking is known to be associated with an increased risk for IPF as well as familial interstitial pneumonia [8]. Moreover, it is also associated with a decrease in WRN protein in lung fibroblasts and cigarette smoke extract induces cellular senescence via WRN downregulation in cultured fibroblast [9]. Our patient had a very high tobacco consumption which started at an unusually young age (10 years old) that probably contributed to the development of ILD.

The changing profile showed that our patient had a slow progression between 2007 and 2012 and a fatal accelerated decline in 2012. No event has been found to be responsible for this decline. Whether WS is a risk factor for rapid decline in lung function is questionable. In a recent series of 9 transplant patients with telomerase mutation associated ILD, 5 patients were transplanted, thanks to a high-emergency allocation rule, after acute exacerbation or accelerated decline [10]. This frequency is higher than in IPF patients without genetic predisposition. The accelerated decline in our patient, as well as in patients with mutation in telomerase complex, could be secondary to a defect in lung repair favor by telomere instability after injury.

Our report has some limits. First of all we have a single case that could be an incidental finding but WS is a very rare disease. We could not perform lung biopsy or postmortem examination, and we did not perform screening of gene encoding SP-C, ABCA3, and TTF1 as well as telomere length measurement. In our opinion, this first association should be brought to the attention of physicians despite those limits.

In conclusion, our case suggests that WS could be associated with pulmonary fibrosis especially in smokers of 50 years of age and over. The recently increasing longevity in WS patients could lead to witnessing more cases of IPF in this context. Overall, early identification of ILD may improve clinical outcome by counselling WS patients regarding smoking cessation and by proposing new therapy available for IPF (i.e., pirfenidone and nintedanib) in order to prevent progression and/or accelerated decline.

## References

[1] Goto M., Ishikawa Y., Sugimoto M., Furuichi Y. (2013). Werner syndrome: a changing pattern of clinical manifestations in Japan (1917–2008). *BioScience Trends*.

[2] Goto M., Matsuura M. (2008). Secular trends towards delayed onsets of pathologies and prolonged longevities in Japanese patients with Werner syndrome. *BioScience Trends*.

[3] Armanios M. (2012). Telomerase and idiopathic pulmonary fibrosis. *Mutation Research*.

[4] Calado R. T., Young N. S. (2009). Telomere diseases. *The New England Journal of Medicine*.

[5] de Leon A. D., Cronkhite J. T., Yilmaz C. (2011). Subclinical lung disease, macrocytosis, and premature graying in kindreds with telomerase (TERT) mutations. *Chest*.

[6] Raghu G., Lynch D., Godwin J. D. (2014). Diagnosis of idiopathic pulmonary fibrosis with high-resolution CT in patients with little or no radiological evidence of honeycombing: secondary analysis of a randomised, controlled trial. *The Lancet Respiratory Medicine*.

[7] Nalysnyk L., Cid-Ruzafa J., Rotella P., Esser D. (2012). Incidence and prevalence of idiopathic pulmonary fibrosis: review of the literature. *European Respiratory Review*.

[8] Rosas I. O., Ren P., Avila N. A. (2007). Early interstitial lung disease in familial pulmonary fibrosis. *American Journal of Respiratory and Critical Care Medicine*.

[9] Nyunoya T., Monick M. M., Klingelhutz A. L. (2009). Cigarette smoke induces cellular senescence via werner's syndrome protein down-regulation. *American Journal of Respiratory and Critical Care Medicine*.

[10] Borie R., Kannengiesser C., Hirschi S. (2015). Severe hematologic complications after lung transplantation in patients with telomerase complex mutations. *The Journal of Heart and Lung Transplantation*.

